# Lenvatinib plus anti-PD-1 therapy represents a feasible conversion resection strategy for patients with initially unresectable hepatocellular carcinoma: A retrospective study

**DOI:** 10.3389/fonc.2022.1046584

**Published:** 2022-11-24

**Authors:** Yong Yi, Bao-Ye Sun, Jia-Lei Weng, Cheng Zhou, Chen-Hao Zhou, Ming-Hao Cai, Jing-Yun Zhang, Hong Gao, Jian Sun, Jian Zhou, Jia Fan, Ning Ren, Shuang-Jian Qiu

**Affiliations:** ^1^ Department of Liver Surgery, Liver Cancer Institute, Zhongshan Hospital, and Key Laboratory of Carcinogenesis and Cancer Invasion (Ministry of Education), Fudan University, Shanghai, China; ^2^ Department of Hepatobiliary Surgery, Chongqing Emergency Medical Center, The Fourth People’s Hospital of Chongqing, Chongqing University, Chongqing, China; ^3^ Key Laboratory of Whole-Period Monitoring and Precise Intervention of Digestive Cancer of Shanghai Municipal Health Commission, and Institute of Fudan-Minhang Academic Health System, Minhang Hospital, Fudan University, Shanghai, China

**Keywords:** hepatocellular carcinoma, lenvatinib, Anti-PD-1 therapy, conversion resection, objective response

## Abstract

**Purpose:**

We aimed to investigate the feasibility of lenvatinib plus anti-PD-1 therapy as a conversion therapy for initially unresectable hepatocellular carcinoma (HCC).

**Methods:**

Patients with initially unresectable HCC who received combined lenvatinib and anti-PD-1 antibody between May 2020 and Jan 2022 in Zhongshan Hospital were retrospectively analyzed. Tumor response and resectability were assessed by imaging every two months according to RECIST version 1.1 and modified RECIST (mRECIST) criteria.

**Results:**

A total of 107 patients were enrolled. 30 (28%) of them received conversion surgery within 90.5 (range: 53–456) days after the initiation of lenvatinib plus anti-PD-1 therapy. At baseline, the median largest tumor diameter of these 30 patients was 9.2 cm (range: 3.5-15.0 cm), 26 patients had Barcelona Clinic Liver Cancer stage B-C, and 4 had stage A. Prior to surgery, all cases displayed tumor regression and 15 patients achieved objective response. Pathological complete response (pCR) was observed in 10 patients. No severe drug-related adverse events or surgical complications were observed. After a median follow-up of 16.5 months, 28 patients survived and 11 developed tumor recurrence. Survival analysis showed patients achieving tumor response before surgery or pCR had a longer tumor-free survival. Notably, patients with microvascular invasion (MVI) had significantly higher recurrence rate and poorer overall survival than patients without.

**Conclusions:**

Lenvatinib combined with anti-PD-1 therapy represents a feasible conversion strategy for patients with initially unresectable HCC. Patients achieving tumor responses are more likely to benefit from conversion resection to access a longer term of tumor-free survival.

## Introduction

Hepatocellular carcinoma (HCC), more than 50% cases of which occurs in China, ranks the sixth most prevalent cancer and the fourth leading cause of cancer death globally (1). Despite the developing technology for detecting early-stage HCC, more than 50% HCC patients are still diagnosed at an advanced stage when the opportunity for radical surgical resection has been already lost, resulting in a poor clinical outcome (2). For these HCC patients, systemic therapies including multi-kinase inhibitors, intra-arterial therapy, chemotherapy, and immunotherapy, are beneficial but still not optimistic (3).

The concept of conversion therapy has been proposed with the aim of downstaging tumor to convert initially unresectable tumor into resectable and improving the long-term survival of patients with advanced tumors. This treatment strategy has also been widely practiced in HCC for more than 30 years, using several locoregional therapies like transcatheter arterial chemoembolization and associating liver partition and portal vein ligation for staged hepatectomy to reduce tumor burden and increase the feasibility of surgery (4–7). However, currently potential conversion regimens still need to be further supported by sufficient clinical evidence to facilitate their routine application in clinical practice.

Systemic therapies for HCC mainly include tyrosine kinase inhibitors (TKIs), immunotherapy and chemotherapy. Traditional sorafenib treatment had a relatively low tumor response rate and thus was not considered as part of conversion therapy for advanced HCC (8–10). Lenvatinib has been approved as a new first-line option for advanced HCCs and is associated with significantly higher overall response rates than sorafenib in unresectable HCC (11, 12). In addition, immune checkpoint inhibitors (ICIs) have proved to have an effective anti-tumor activity in advanced HCC patients. Furthermore, pre-clinical and clinical studies prove that lenvatinib plus pembrolizumab has higher objective response rate (ORR) than single-agent treatment, and is one of the most promising therapies among all the regimen of combinations (13–16). Notably, a small sample study from our institution has reported that anti-PD-1 therapy combined with TKIs can improve the rate of conversion resection in initially unresectable HCC (17). Thus, advances in systemic therapy, represented by lenvatinib combined with anti-PD-1 therapy, have led clinicians to reassess the value of systemic therapy in the conversion therapy of HCC.

Herein, we report 30 initially unresectable HCC patients who received lenvatinib and anti-PD-1 combination therapy, followed by successful surgical resection. To the best of our knowledge, this study represents the largest cohort of HCC patients undergoing conversion rection following systemic therapy. Furthermore, we performed survival analysis of the 30 patients and revealed the association between patient clinical outcomes and treatment response as well as microvascular invasion (MVI). Our study provides clinical evidence to evaluate the efficacy and safety of systemic therapy for HCC in the conversion therapy setting.

## Materials and methods

### Study design and patient population

A total of 107 patients with unresectable or advanced HCC who received lenvatinib with anti-PD-1 antibodies at Zhongshan Hospital, Fudan University between May 2020 and Jan 2022 were retrospectively analyzed in this study. The inclusion criteria comprised the following (1): Clinically confirmed HCC based on the domestic guideline and American Association for the Study of Liver Diseases criteria (18, 19) (2); Tumor was considered unresectable either due to intermediate to advanced HCC or insufficient postoperative remnant liver volume (3); At least one measurable tumor lesion according to modified Response Evaluation Criteria in Solid Tumors (mRECIST) (20). The presence of vascular invasion was diagnosed using the Magnetic Resonance Imaging (MRI). The clinical data of these patients were obtained from the medical record system. This study was approved by the Ethics Committee of Zhongshan Hospital and conducted conformed to the standards set by the Declaration of Helsinki. All patients signed the written informed consent before systemic therapy or surgery.

### Systemic therapy

Systemic therapy included lenvatinib plus intravenous anti-PD-1 antibody. Lenvatinib was orally administered once daily (body weight ≥60 kg, 12 mg; <60 kg, 8 mg; Eisai, Inc., Tokyo, Japan). Anti-PD-1 antibody like camrelizumab (Hengrui Medicine, Jiangsu, China) 200 mg (21), was administered every two weeks, and sintilimab (Innovent Biologics, Suzhou, China and Eli Lilly and Company, Inc., Indianapolis, IN, USA) 200 mg (22), or toripalimab (Junshi Bioscience, Shanghai, China) 240 mg (23), or pembrolizumab (MSD, State of New Jersey, USA) 200 mg, or tislelizumab (BeiGene, Beijing, China) 200 mg (24), was administered every three weeks. During the study period, all anti-PD-1 antibodies were off-label regimens for HCC and could not be covered by the medical insurance system in China. Thus, anti-PD-1 antibodies were utilized based on patient preference, mainly due to the economic cost and the updated information from ongoing clinical trials. Patients were monitored for hematuria routine, tumor markers, liver, kidney, thyroid, adrenal and cardiac functions every 2-3 weeks prior to the anti-PD-1 antibody treatment.

### Treatment response evaluation

Tumor responses were evaluated using abdominal MRI and chest computed tomography (CT) every two months, and determined according to the Response Evaluation Criteria in Solid Tumors (RECIST) v1.1 (25) and mRECIST (20). Both investigator review (INR) and independent imaging review (IIR) were conducted for all evaluation. Briefly, complete response (CR) was defined as a complete disappearance of all tumor lesions, partial response (PR) as the sum of tumor diameters were reduced by ≥ 30% from baseline, progressive diseases (PD) as the sum of tumor diameters were increased by ≥ 20% from baseline, and stable diseases (SD) as neither CR, PR nor PD. The ORR was calculated as the sum of percentage of patients who achieved CR and PR. All image processing and measurement were performed by two independent radiologists, who were blinded to the patient information. Safety evaluations were performed according to the Common Terminology Criteria for Adverse Events version 4.03.

### Surgical resection procedure

Tumor resectability was assessed based on the imaging results with the following criteria (1): R0 resection could be achieved with residual liver volume more than 35% and no contraindications to hepatic resection (2); intrahepatic lesions were assessed as CR, PR, or regressed SD without severe drug-related adverse events (AEs). The surgical procedure was conducted as previously reported (26). Tumor resectablility was discussed and passed by the Multidisciplinary Team (MDT) composed of senior surgeons and radiologists in Zhongshan Hospital. Completely resected tumor samples without residual viable tumor cells after hematoxylin and eosin staining were considered as pathological complete response (pCR). Combination therapy was resumed 4 weeks after surgery and follow-up imaging and serum tumor biomarkers were performed every 2-3 months. Overall survival (OS) was determined as the interval between the date of surgery and the date of death or study endpoint. Recurrence-free survival (RFS) was defined as the interval between the date of surgery and the date of tumor recurrence or study endpoint.

### Statistical analysis

Statistical analyses were conducted using SPSS v.25 (IBM Inc., Armonk, NY, USA). Categorized variables were summarized as frequencies (proportion). The value and 95% confidence interval (CI) of ORR was calculated by the Clopper-Pearson algorithm. Survival differences were analyzed by the Kaplan-Meier method. Two-sided P value < 0.05 was considered statistically significant.

## Results

### Baseline characteristics of the study cohort

**Figure 1:**
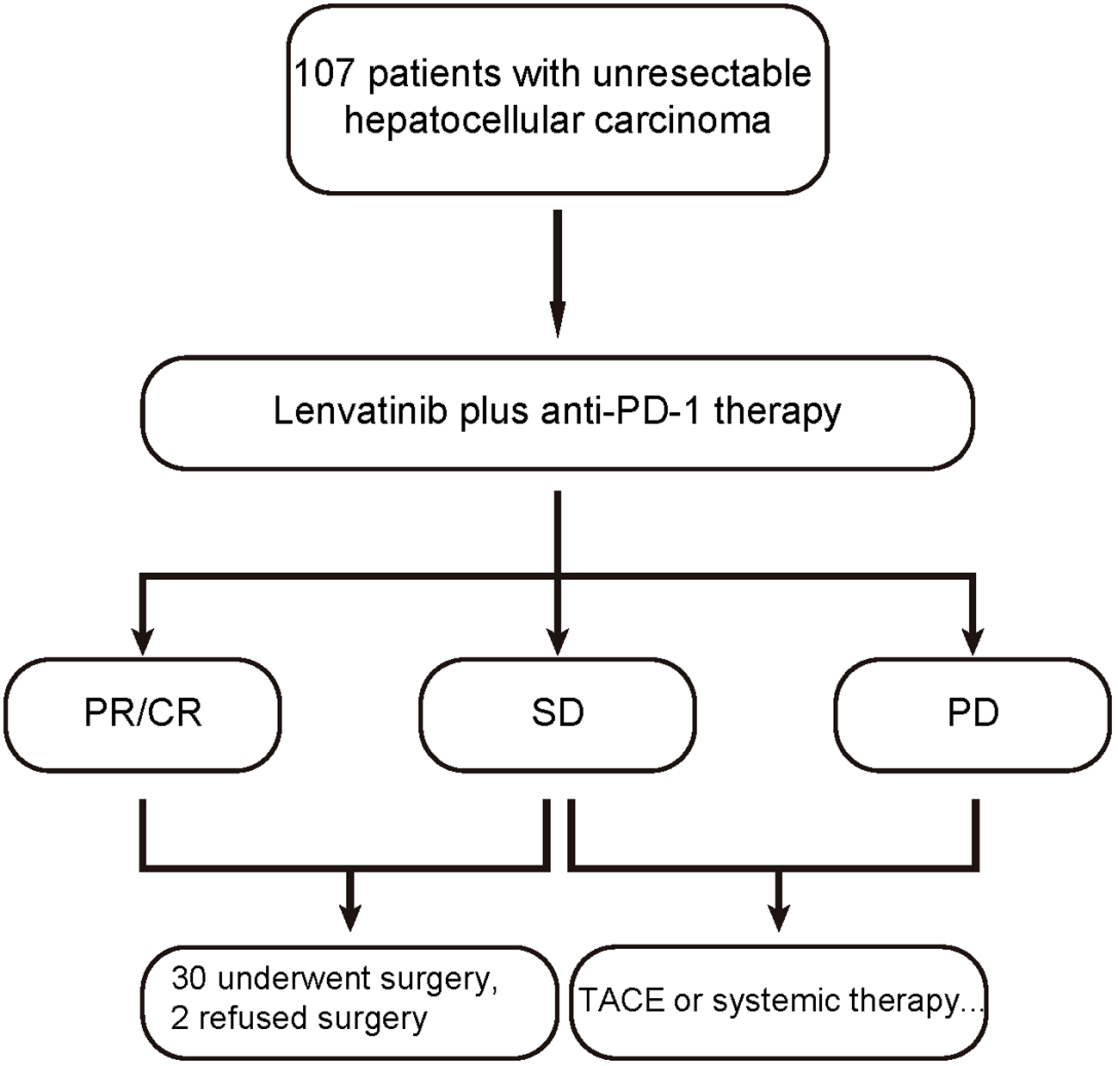
Patient flowchart.

From May 2020 to Jan 2022, 107 consecutive HCC patients who received lenvatinib plus anti-PD-1 antibodies as first-line systemic therapy were retrospectively analyzed. Of these patients, 32 were evaluated as resectable and 30 (30/107, 28%) successfully underwent R0 conversion resection. Other 2 patients refused surgery and continued to receive the systemic therapy (**Figure 1**). The baseline demographic and clinical characteristics of the 30 patients are summarized in **Table 1**. Briefly, the median age of these patients was 56.0 (range: 40–70) years, and 83.3% of them were male. As for Eastern Cooperative Oncology Group (ECOG) performance status, 63.3% of them had a score of 0. Twenty-four (80%) patients had an etiology of chronic HBV infection. Twenty (20/30, 66.7%) patients had macrovascular invasion, including portal vein or hepatic vein tumor thrombus in 19 patients and intrahepatic bile duct tumor thrombus in 1 patient. Four patients had Barcelona Clinic Liver Cancer (BCLC) stage A disease but were evaluated as unresectable, consisting of 2 patients due to anatomical constraints removing the tumor, and 2 patients with insufficient functional hepatic reserve.

**Table 1:** Baseline demographic and clinical characteristics of HCC patients receiving surgery after treatment with lenvatinib plus anti-PD-1 antibody (n = 30).

Characteristics	Number (%)
Age, years	
Median	56
Range	40-70
Gender	
Male	25 (83.3%)
Female	5 (16.7%)
ECOG PS score	
0	19 (63.3%)
1	11 (36.7%)
HBsAg	
Positive	24 (80.0%)
Negative	6 (20.0%)
Alpha-fetoprotein, ng/mL	
≤ 300	17 (56.7%)
> 300	13 (43.3%)
PIVKA-II, mAU/mL	
≤ 1000	5 (16.7%)
> 1000	25 (83.3%)
Macrovascular invasion^a^	20 (66.7%)
BCLC stage	
0-A	4 (13.3%)
B-C	26 (86.7%)
CNLC stage	
I a	1 (3.3%)
I b	3 (10.0%)
II b	6 (20.0%)
III a	17 (56.7%)
III b	3 (10.0%)

BCLC, Barcelona Clinic Liver Cancer; CNLC, China National Liver Cancer; ECOG PS, Eastern Cooperative Oncology Group Performance Status; HBsAg, Hepatitis B surface antigen; HCC, Hepatocellular carcinoma; PD-1, Programmed death receptor 1; PIVKA-II, protein induced by vitamin K absence-II.

^a^Indicators based on radiographic evidence.

### Treatment efficacy of lenvatinib combined with anti-PD-1 therapy

**Figure 2:**
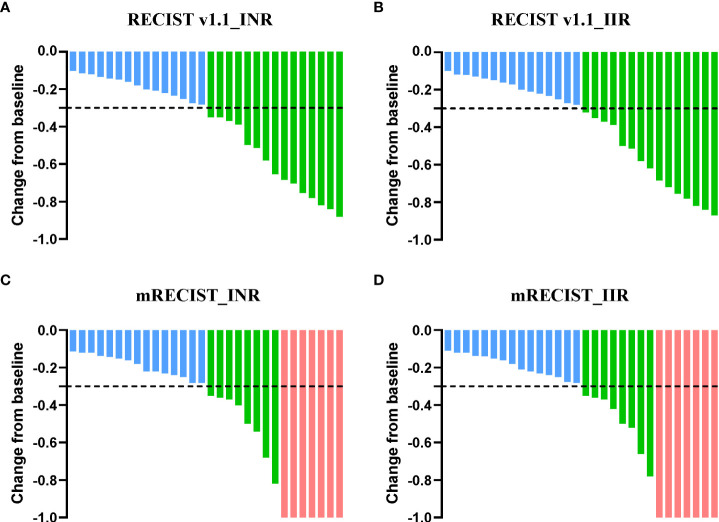
Percent change of the sum of tumor diameters from baseline. Percent change of total tumor diameters from baseline by INR **(A)** and IIR assessment **(B)** according to RECIST Version 1.1. Overall percent change from baseline in tumor size by INR **(C)** and IIR assessment **(D)** according to mRECIST criteria. Each bar represents one patient. INR, Investigator review; IIR, Independent imaging review; mRECIST, modified response evaluation criteria in solid tumors.

Tumor responses were evaluated *via* preoperative radiographic imaging (**Table 2**). According to the criteria of RECIST Version 1.1, the ORR was 50% (95% CI, 31.3%-68.7%) by both INR and IIR assessment. PR was achieved in 15 patients (50%) and SD was observed in the rest of the patients. By mRECIST criteria, 7 (23.3%), 8 (26.7%), and 15 (50%) patients had CR, PR, and SD respectively. All cases displayed tumor regression and a decrease in tumor size compared with that at baseline (**Figure 2**).

**Table 2:** Efficacy of lenvatinib plus anti-PD-1 antibody in patients receiving surgery (n = 30).

Response	RECIST Version 1.1		mRECIST	
	INR	IIR	INR	IIR
Objective response rate, %	50.0	50.0	50.0	50.0
95% CI^a^	31.3-68.7	31.3-68.7	31.3-68.7	31.3-68.7
Complete response	0 (0%)	0 (0%)	7 (23.3%)	7 (23.3%)
Partial response	15 (50.0%)	15 (50.0%)	8 (26.7%)	8 (26.7%)
Stable disease	15 (50.0%)	15 (50.0%)	15 (50.0%)	15 (50.0%)

IIR, Independent imaging review; INR, Investigator review; mRECIST, modified response evaluation criteria in solid tumors.

a Calculated using the Clopper-Pearson method.

### Surgery and perioperative findings

The median diameter of the major liver tumor was 9.2 cm (range: 3.5–15 cm), and 5 patients had multiple intrahepatic lesions (**Table 3**). The median interval from the initiation of systemic therapy to surgery was 90.5 (range: 53–456) days. All patients received at least 3 infusions of anti-PD-1 antibodies, with a median infusion of 4 (range: 3–21) times. All patients underwent open surgery. The median time of surgery was 180 (range: 150–220) minutes, and the median volume of blood loss was 400 (range: 100–1500) milliliters. Three patients received intraoperative blood transfusion. The median postoperative hospital stay was 14 (range: 9–31) days. All patients recovered well and no major postoperative complications occurred. A total of 10 patients (10/30, 33.3%) achieved a pathological complete response (pCR) in all surgical specimens, and MVI was detected in 8 (8/30, 26.7%) patients (**Table 3**).

**Table 3:** Surgical and pathological features of patients receiving conversion surgery (n = 30).

Characteristics	Total (n = 30)
Days from systemic therapy to surgery, days (median, range)	90.5 (53–456)
Anti-PD-1 antibody used	
Sintilimab	14 (46.7%)
Toripalimab	8 (26.7%)
Tislelizumab	4 (13.3%)
Camrelizumab	3 (10%)
Pembrolizumab	1 (3.3%)
Infusions of anti-PD-1 antibody, times (median, range)	4 (3-21)
Major tumor size, cm (median, range)	9.3 (3.5-15)
Tumor number	
Solitary	25 (83.3%)
Multiple	5 (16.7%)
Open surgery, n (%)	30 (100%)
Time of surgery, mins (median, range)	180 (150–220)
Blood loss, mL (median, range)	400 (100-1500)
Blood transfusion	3 (10.0%)
Postoperative hospital stay, days (median, range)	14 (8-31)
Micro-vascular invasion, n (%)	8 (26.7%)
Pathological complete response, n (%)	10 (33.3%)

**Figure 3:**
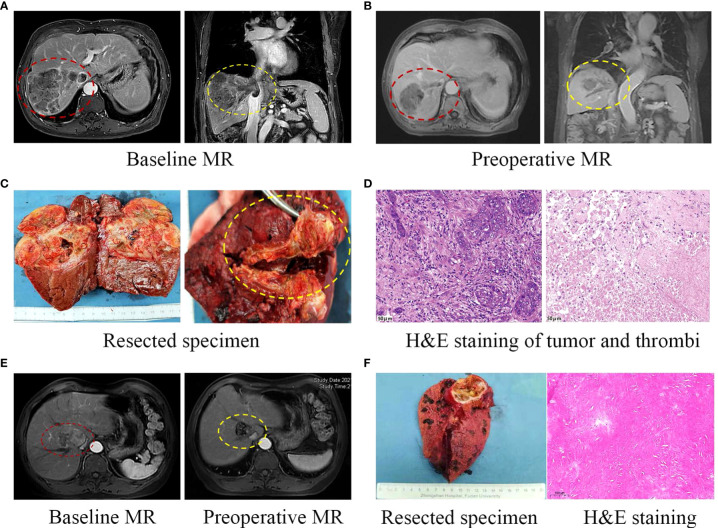
Two representative cases. **(A)** Pre-treatment MR showing both the liver tumor and macrovascular tumor thrombi. **(B)** Preoperative MR showing the regression of liver tumor and tumor thrombi following systemic therapy. **(C)** Curative resection of liver tumor and tumor thrombi in right hepatic vein and inferior vena cava. **(D)** HE staining of surgically resected tumor samples (left) and complete necrosis of the macrovascular tumor thrombi with infiltration of inflammatory cells (right, 200×). **(E)** This case was classified as BCLC stage A, with tumor compression on hepatic vein and inferior vena cava. After the combination therapy, obvious regression of the tumor was observed. The patient underwent curative liver resection and HE staining of resected tumor samples showed massive necrosis without viable tumor cells **(F)**.

Two representative cases were shown in **Figure 3**. Patient 8 was diagnosed with BCLC stage C HCC, with tumor invading into the right hepatic vein and inferior vena cava (**Figure 3A**). 12 weeks after the treatment with lenvatinib plus tislelizumab, MR scans showed significant necrosis in both the tumor and tumor thrombi (**Figure 3B**). Massive necrosis of tumor cells and infiltration of inflammatory cells were observed in the resected tumor samples. Moreover, complete necrosis of the macrovascular tumor thrombi observed by pathological study indicated a downstaging from BCLC stage C to stage A (**Figures 3C, D**). Patient 17 was classified as BCLC stage A, while the tumor compression on nearby hepatic vein and the first porta hepatis, and its proximity to inferior vena cava increased the risk for complete resection, making it surgically unresectable. After the combination therapy with lenvatinib plus toripalimab for 18 weeks, obvious tumor shrinkage and thickening of tumor encapsule decreased the risk of severe bleeding and tumor dissemination during resection, offering this patient an opportunity for surgery (**Figures 3E, F**).

**Figure 4:**
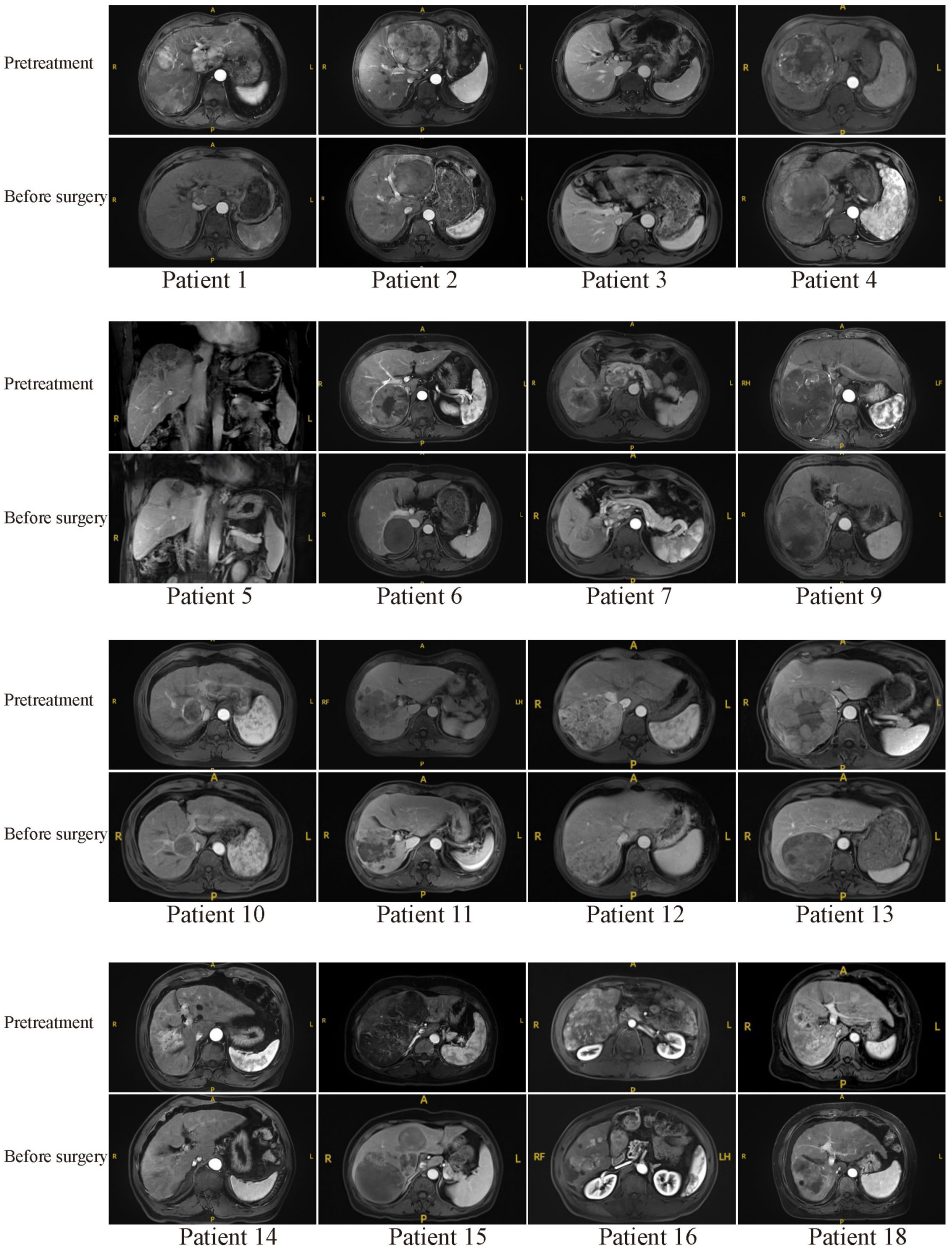
Pretreatment and preoperative MR scans of patients 1-18 (except patients 8 and 17).

**Figure 5:**
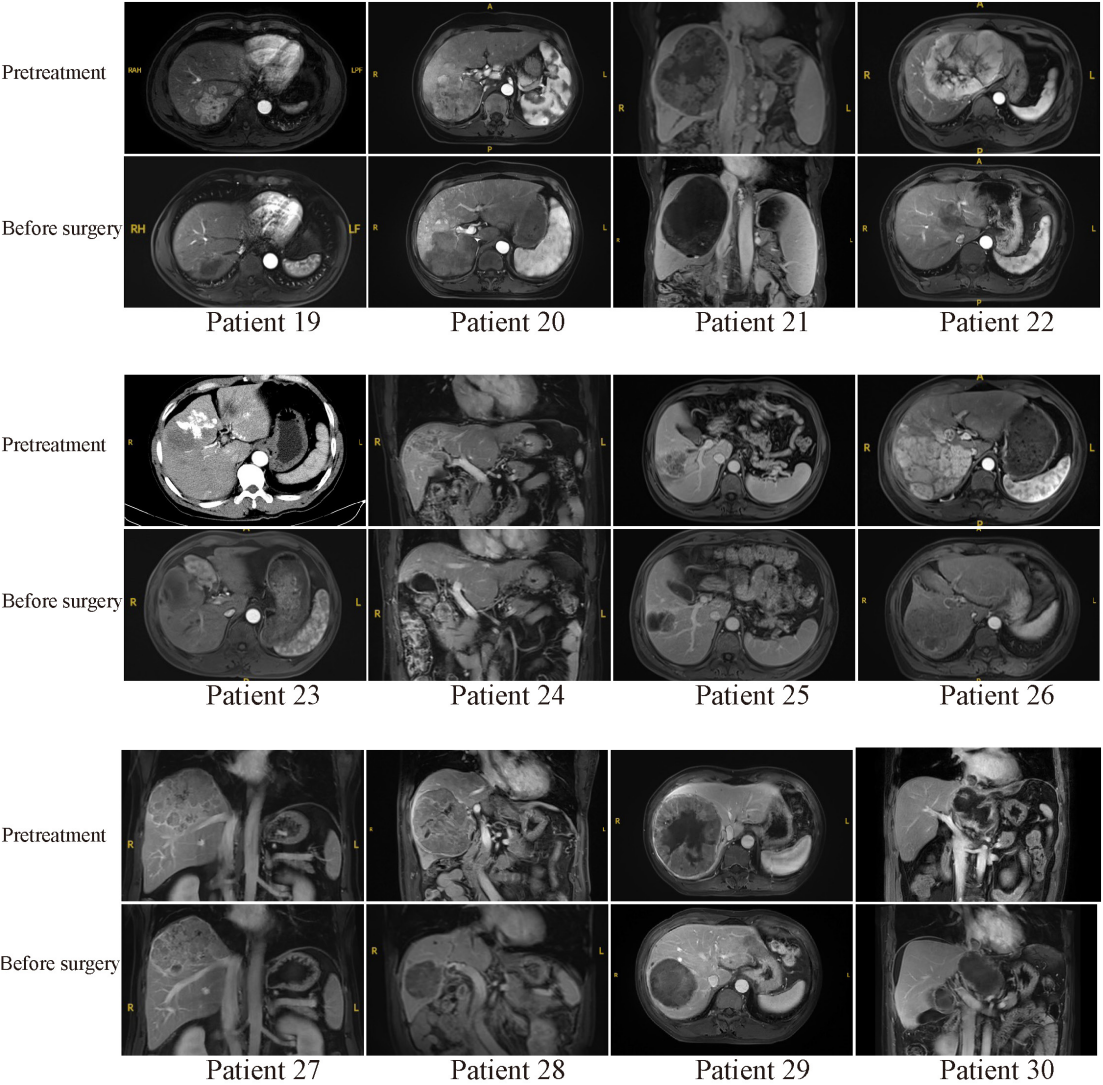
Pretreatment and preoperative MR or CT scans of patients 19-30. Patient 23 received emergency hepatic arterial chemoembolization due to rupture of hepatocellular carcinoma in other hospital before combination therapy.

Typical MR or CT imaging scans of other patients (besides patients 8 and 17) before systemic treatment and before surgery were also listed in **Figures 4**, **5**. The major reason for unresectability were tumor invasion into major portal vein (patients 12, 18 and 22) and the main branches of the portal vein (patients 2, 7, 9, 11, 14, 20, 24, 25, 26, 27 and 28), into hepatic vein or inferior vena cava (patients 5, 21, 29 and 30), into right hepatic bile duct (patient 3), multiple intrahepatic lesions (patients 1, 6, 15, 16, 19 and 23), insufficient remnant liver volume (patients 4 and 13), anatomical constraints for curative resection (patient 10), concomitant hilar or retroperitoneal lymph node metastasis (patients 7, 20 and 21). Obvious tumor regression was observed in all these cases before surgery.

### Safety

The treatment-related AEs were summarized in **Table 4**. Most patients experienced mild, tolerable, and controllable AEs. The most common AEs of any grade were decreased platelet count (n = 14, 46.7%), proteinuria (n = 10, 33.3%), decreased white blood cell count (n = 8, 26.7%), hypertension (n = 6, 20.0%), hypothyroidism (n = 5, 16.7%), and palmar-plantar erythrodysesthesia syndrome (n = 4, 13.3%).

**Table 4:** Treatment-related AEs in patients receiving lenvatinib plus anti-PD-1 antibody (n = 30).

Preferred AE Term	No. (%)			
	Any Grade^a^	Grade 1	Grade 2	Grade 3
Decrease in platelet count	14 (46.7%)	5 (16.7%)	4 (13.3%)	5 (16.7%)
Proteinuria	10 (33.3%)	1 (3.3%)	6 (20.0%)	3 (10.0%)
Decrease in white blood cell count	8 (26.7%)	2 (6.7%)	3 (10.0%)	3 (10.0%)
Hypertension	6 (20.0%)	0	4 (13.3%)	2 (6.7%)
Hypothyroidism	5 (16.7%)	3 (10.0%)	0	2 (6.7%)
Palmar-plantar erythrodysesthesia syndrome	4 (13.3%)	1 (3.3%)	1 (3.3%)	2 (6.7%)
Increased transaminase	4 (13.3%)	0	2 (6.7%)	2 (6.7%)
Weight decreased	3 (10.0%)	0	3 (10%)	0
Hypoadrenalism	3 (10.0%)	0	0	3 (10.0%)
Myocarditis	3 (10.0%)	2 (6.7%)	0	1 (3.3%)
Gastrointestinal bleeding	2 (6.7%)	0	0	2 (6.7%)
Hyperthyroidism	2 (6.7%)	2 (6.7%)	0	0
Abdominal distension	2 (6.7%)	2 (6.7%)	0	0

^a^Adverse events were graded in accordance with the Common Terminology Criteria for Adverse Events v4.0.

### Follow-up and survival analysis

**Figure 6:**
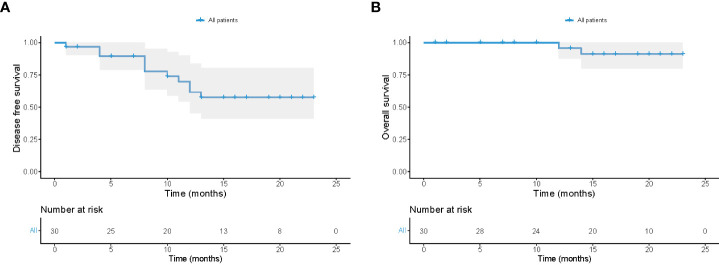
Follow-up of all 30 patients receiving conversion surgery. Kaplan-Meier plots of disease-free survival **(A)** and overall survival **(B)** in all 30 patients.

The cutoff date for the present analysis was July 1, 2022. After a median follow-up of 16.5 months since the date of surgery, 28 patients remained alive and 11 patients developed tumor recurrence (**Figure 6**). All surviving patients continued the combination therapy, with a median time of 12 months. 10 (10/30, 33.3%) patients had a dose reduction of lenvatinib due to its related toxicities. The 12-month overall survival rate of the 30 patients after surgery was 95.7% (standard error, 4.3%), and 12-month disease-free survival rate after surgery was 61.6% (standard error, 9.6%).

**Figure 7:**
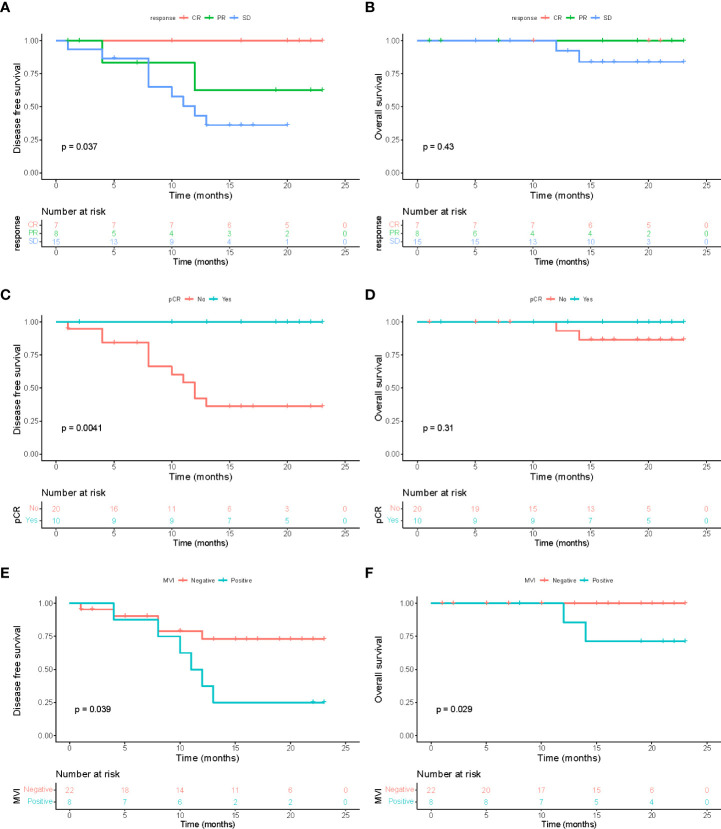
Survival analysis. Disease-free survival **(A)** and overall survival **(B)** among patients with different treatment response to combinational therapy evaluated *via* preoperative MR imaging (mRECIST). Disease-free survival **(C)** and overall survival **(D)** between patients who did and did not achieve a pathological complete response (pCR) in all resected specimens. Disease-free survival **(E)** and overall survival **(F)** between patients with and without microvascular invasion (MVI) in resected tumor samples.

Postoperative survival analysis demonstrated that there was a significant difference in DFS between patients who did and did not achieve a treatment response before surgery (**Figure 7A**; P = 0.037). Compared with patients with SD, patients who achieved CR or PR had a longer DFS, while no statistically significant difference in OS was observed among patents with and without tumor response (**Figure 7B**; P = 0.43). Similar results were found when performing survival analysis between patients who achieved pCR in resected samples and those did not (**Figures 7C, D**; P=0.0041 for DFS, P=0.31 for OS). Notably, patients with the presence of MVI in resected tumor specimens had significantly higher recurrence risk and poorer overall survival than patients without after surgery (**Figures 7E, F**; both P <0.05). This indicated that MVI still acts as a vital prognostic risk factor for HCC patients who received conversion surgery.

## Discussion

Our present study showed that lenvatinib plus anti-PD-1 antibodies allowed successful R0 resection in 28% (30/107) of patients with initially unresectable HCC, indicating this combination therapy is a feasible conversion strategy for converting HCC patients from unresectable to resectable.

Conversion resection aims to achieve downstaging of tumors and allow patients with advanced or initially unresectable cancers to obtain curative resectability. The role of conversion therapy in HCC patients has long been neglected due to lack of effective systemic therapies. Recently, systemic therapeutic agents especially the combination of TKIs with ICIs have shown promising anti-tumor efficacy, reviving the idea of conversion therapy in HCC. Several studies have explored the feasibility of different combination therapies as conversion therapy in HCC. A previous study from our liver cancer institute reported that 15.9% (10/63) of patients with initially unresectable HCC received conversion surgery after combination treatment with TKI and anti-PD-1 antibodies (17). The combination of TKIs, ICIs and local regional therapy has been reported to convert 9 (9/38, 23.7%) HCC patients with extrahepatic metastases from unresectable to resectable (27). A multi-center retrospective study showed that the ORR of triple combination therapy with lenvatinib, toripalimab plus hepatic arterial infusion chemotherapy (HAIC) was as high as 67.6%, and its conversion resection rate was higher than that of lenvatinib alone (12.7% vs 0) (28). In our study, lenvatinib plus PD-1 inhibitors led to an ORR of 50% (15/30) in HCC patients who underwent conversion surgery and the conversion resection rate was 28% (30/107). Notably, the treatment efficacy of the combination therapy could still be underestimated because surgical resection was considered for patients who achieved tumor regression whenever feasible. For example, patients with regressed SD could regress to PR if they continued the systemic therapy rather than surgery. No major postoperative adverse events occurred in these cases. Therefore, lenvatinib plus PD-1 inhibitors can result in downstaging and increase the likelihood of surgical resection in advanced HCC patients, with high potential to prolong their survival.

Postoperative follow-up of the 30 patients revealed that the one-year overall survival rate was 95.7% and the one-year disease-free survival rate was 61.6%, highlighting the significance of surgical resection in improving survival outcomes. Remarkably, while all these patients displayed tumor shrinkage and underwent conversion surgery, patients achieving CR or PR before surgery had a significantly longer DFS compared with those with SD. The pCR rate was 33.3% (10/30) compared with 60% (6/10) reported before (17) and patients who achieved pCR also had better DFS than those did not. These results suggested that patients with tumor responses, whether evaluated by preoperative imaging or by pathological findings of resected specimens, are more likely to benefit from conversion resection to access a longer term of tumor-free survival.

Tumor recurrence often occurs because micro-metastasis persists even after curative resection. The presence of MVI has been determined to accurately predict the early recurrence and adverse clinical outcomes of HCC patients undergoing liver resection (29–32). In this study, pre-treatment MR scans showed that 20 (20/30, 66.7%) patients had macrovascular invasion, which was not recommended for resection by BCLC guideline. After surgery, MVI was detected in 8 (8/30, 26.7%) resected tumor samples, compared with the MVI incidence of 63% with tumor size larger than 6.5cm reported previously (33), suggesting that lenvatinib plus PD-1 inhibitors could effectively eradicate microvascular tumor thrombi and decrease the positive rate of MVI. Moreover, patients with MVI had significantly shorter tumor-free survival and OS than patients without MVI. This indicated that MVI retained its vital prognostic value in patients receiving conversion surgery. Surgical resection, followed by adjuvant intervention therapy and systemic therapy, could be utilized to reduce the burden of MVI and prolong the survival of this subgroup of patients with MVI, although further investigation is warranted in real world studies.

Safety evaluations revealed that there were no severe (grade 4 or 5) treatment-related adverse events in these patients during the systemic treatment period. Most treatment-related AEs were mild and tolerable, indicating that the drug toxicity can be well addressed and controlled by dynamic monitoring and dose modification in the clinical practice (15).

Several limitations of this study should be noted. First, our study enrolled a relatively small number of HCC patients from a single center although it represented the largest reported cohort of HCC patients receiving conversion surgery so far. The occurrences of adverse events were mainly evaluated according to the blood tests and medical records, thus could be underestimated due to the retrospective nature of our study. Second, the patients in our study were heterogeneous regarding to the regimens of anti-PD-1 monoclonal antibody while no evidence has shown the different effects of these anti-PD-1 antibodies. Thus, this strategy needs prospective validation in a more consistent and large-scale multicenter study in the future.

In conclusion, lenvatinib plus PD-1 inhibitors exhibited promising anti-tumor activity with manageable toxicity. More importantly, this combination treatment strategy results in tumor downsizing and allows patients with unresectable HCC to access surgical resection, with high potential to prolong their long-term survival. Treatment response and MVI status could predict the survival outcomes, especially the tumor-free survival of patients receiving conversion surgery.

## Data availability statement

The original contributions presented in the study are included in the article/Supplementary Material. Further inquiries can be directed to the corresponding authors.

## Ethics statement

The studies involving human participants were reviewed and approved by The Ethics Committee of Zhongshan Hospital. The patients/participants provided their written informed consent to participate in this study.

## Author contributions

Study concept, design, and supervision (S-JQ, NR, JZ, JF), analysis and interpretation of data (YY, B-YS), drafting of the manuscript (B-YS, J-LW, M-HC), acquisition of data (YY, B-YS, CZ), preparation of figures and tables (J-LW, HG), critical revision of the manuscript for important intellectual content (C-HZ, JS), provision of patient tissue samples (CZ, J-YZ). All authors contributed to the article and approved the submitted version.

## Funding

The work was supported by the National Natural Science Foundation of China (82072672, 81772510, 82073208, 82103521, 82072677); the Clinical Research Project of Zhongshan Hospital (2020ZSLC62, 2020ZHZS17); the Research and Development Program of Zhongshan Hospital (2019ZSFZ24); the Shanghai Sailing Program (21YF1407500); and the China Postdoctoral Science Foundation (2021M690674).

## Acknowledgments

We thank the patients for participating in this study and their families.

## Conflict of interest

The authors declare that the research was conducted in the absence of any commercial or financial relationships that could be construed as a potential conflict of interest.

## Publisher’s note

All claims expressed in this article are solely those of the authors and do not necessarily represent those of their affiliated organizations, or those of the publisher, the editors and the reviewers. Any product that may be evaluated in this article, or claim that may be made by its manufacturer, is not guaranteed or endorsed by the publisher.

## Abbreviations

AEs, Adverse events; BCLC, Barcelona Clinic Liver Cancer; CNLC, China Liver Cancer; CR, Complete response; ECOG PS, Eastern Cooperative Oncology Group Performance Status; HBsAg, Hepatitis B surface antigen; HCC, Hepatocellular carcinoma; ICIs, Immune checkpoint inhibitors; IIR, Independent imaging review; INR, Investigator review; mRECIST, Modified response evaluation criteria in solid tumors; ORR, Objective response rate; OS, overall survival; PD, Progressive diseases; PR, Partial response; SD, Stable disease; TKI, Tyrosine kinase inhibitors.
